# The prognostic role of diet quality in patients with MAFLD and physical activity: data from NHANES

**DOI:** 10.1038/s41387-024-00261-x

**Published:** 2024-02-23

**Authors:** Jiaofeng Huang, Yinlian Wu, Jiaping Zheng, Mingfang Wang, George Boon-Bee Goh, Su Lin

**Affiliations:** 1 Department of Hepatology, Hepatology Research Institute, the First Affiliated Hospital, Fujian Medical University, Fuzhou, Fujian China; 2 Fujian Clinical Research Center for Hepatopathy and Intestinal Diseases, Fuzhou, China; 3 Department of Hepatology, National Regional Medical Center, Binhai Campus of the First Affiliated Hospital, Fujian Medical University, Fuzhou, Fujian China; 4 Department of Rehabilitation Medicine, School of Health, Fujian Medical University, Fuzhou, China; 5 Department of Gastroenterology & Hepatology, Singapore General Hospital, Singapore, Singapore; 6 Duke-NUS Medical School, Singapore, Singapore

**Keywords:** Metabolic syndrome, Obesity

## Abstract

**Background and objectives:**

Dietary control and increased physical activity (PA) are recommended for patients with metabolic (dysfunction-) associated fatty liver disease (MAFLD). However, not all patients can sustain both exercise and a healthy diet. This study explored the interaction between dietary quality, PA levels, and mortality in MAFLD patients.

**Methods:**

The Third National Health and Nutrition Examination Survey and linked mortality data were used in this study. Diet quality was assessed with the Healthy Eating Index (HEI). PA level was calculated by multiply self-reported exercise frequency and its Metabolic Equivalent A high-quality diet was associated. A Cox proportional hazard model was used to explore risk factors for mortality in MAFLD patients.

**Results:**

In total, 3709 participants with MAFLD were included in the final analysis. The median follow-up time was 26.2 (interquartile range 19.3–28.1) years and 1549 (41.8%) deaths were recorded over follow-up. Cox multivariate regression was used to adjust for potential confounders of mortality. The results showed both HEI score and PA level were inversely correlated with all-cause mortality (*P* < 0.05). In the subgroup analysis stratified by PA level, higher diet quality decreased all-cause mortality, cardiovascular-related mortality and cancer-related mortality in PA inactive of MAFLD patients (*P* < 0.05), but these correlations were not present in active PA groups.

**Conclusion:**

Healthy diet and physical activity may have different impact as lifestyle interventions for MAFLD. A high-quality diet is associated less mortality in inactive individuals with MAFLD but not in those with active PA levels. Sedentary individuals require healthier diet.

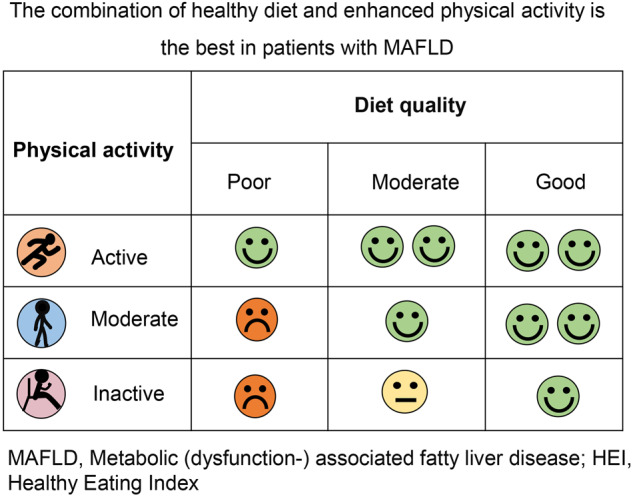

## Introduction

Metabolic (dysfunction-) associated fatty liver disease (MAFLD), previously called non-alcoholic fatty liver disease (NAFLD), is characterized by the presence of metabolic dysfunction and excessive accumulation of triglycerides in the liver [1]. MAFLD has been associated with an increased risk of mortality [2–4].

Currently, there are no approved pharmacological treatments for MAFLD. Lifestyle modification, including dietary control and increased physical activity (PA), remains the first-line intervention [1, 5]. Combined diet and exercise strategies have been more effective in reducing lipid accumulation and improving histology [1].

However, it is difficult for many people to sustain exercise routines. There are many barriers to exercise in patients with fatty liver disease, including physiologic, psychological, and socio-environmental factors [6, 7]. A recent study revealed lower compliance with the Physical Activity Guidelines for Americans in individuals with fatty liver disease than in those without [8].

Low dietary quality is another independent risk factor of NAFLD-related liver mortality [9]. Diet quality can be evaluated using the Healthy Eating Index (HEI), a quantitative score developed by the United States Department of Agriculture to measure the overall quality of people’s diets [10]. It remains unclear how the interaction between diet quality and PA levels might influence the MAFLD outcomes. In this study, we used publicly accessed longitudinal data to explore the association between diet quality, PA levels, and mortality in MAFLD patients, particularly in physically inactive individuals.

## Methods

### Data source

The current study used the Third National Health and Nutrition Examination Survey (NHANESIII, 1988–1994) and linked mortality data from 2019. The NHANES is a nationally representative, cross-sectional survey designed by the National Center for Health Statistics to assess the health and nutritional status of non-institutionalized individuals in the United States. NHANES data are publicly available at https://www.cdc.gov/nchs/nhanes/about_nhanes.htm. Participants were followed until 31 December 2015, and the linked mortality file was made available online.

### Ultrasound and hepatic steatosis

Ultrasonography was performed to assess hepatic steatosis. NHANES III had two ultrasound tests for hepatic steatosis, and we integrated these two datasets. The degree of hepatic steatosis, as assessed by ultrasonography, was categorized as normal (no steatosis), mild, moderate, or severe. In this study, the latter three were regarded as hepatic steatosis.

### Physical activity

Participants were asked about the frequency and type of the following exercises performed in the past month: walking, jogging or running, riding a bicycle, swimming, aerobic or other dance, calisthenic or floor exercise, gardening or yard work, weightlifting, and other activities. The intensity of each activity was presented as metabolic equivalent (MET) and provided in the original dataset. Because the exact duration of each exercise session was not defined in the survey, we were not able to convert exercise activity into metabolically equivalent tasks (MET·min·week) [11]. To evaluate the PA levels, we multiplied the self-reported exercise frequency of activity and its MET to get an activity score. Then the study population was divided into PA active and inactive groups according to the median value of total activity score.

### Dietary quality

The NHANES database provided the HEI score of each participant to estimate the overall dietary quality [12]. HEI scores were assessed using a 24-h dietary recall.

### Income

Income was assessed based on the poverty income ratio, an index reflecting the ratio of household income to household poverty level, determined by the area of residence and household size. Low family income was defined as a ratio of below 1 [13].

### Education

In NHANES III, education was collected as a numerical variable (number of years of education) from 0 to 17 years. A low educational level was defined as an education of <12 years [13].

### MAFLD

MAFLD was diagnosed according to the new definition from an international expert consensus statement for MAFLD [14], which included evidence of ultrasonography-confirmed hepatic steatosis with any of the following three conditions: overweight/obesity, type 2 diabetes, or at least two metabolic risk factors in non-obese individuals. Liver fibrosis was determined using two noninvasive markers: fibrosis 4 index (FIB-4) [15] and NAFLD fibrosis score (NFS) [16].

### Other indicators

The waist-to-hip ratio (WHR) was defined as the waist circumference divided by hip circumference. Body mass index (BMI) was defined as body weight divided by the square of height. Routine laboratory examinations, including glycosylated hemoglobin (HbA1c), alanine aminotransferase (ALT), aspartate aminotransferase (AST), serum cholesterol, and triglycerides, were all collected from the dataset. Detailed technical descriptions of the laboratory methods are available on the NHANES website. Estimated glomerular filtration rate (eGFR) was calculated according to the 2009 CKD Epidemiology Collaboration formula [17].

### Statistical analysis

Quantitative variables were expressed as mean ± standard deviation or median with interquartile range and compared using the Student’s t-test or Mann–Whitney U-test. Qualitative variables were expressed as counts (percentages) and compared using the *χ*^*2*^ test. The Cox proportional hazard model was used to explore the association between HEI and prognosis in MAFLD patients. In this study cohort, individuals of the death groups were significantly older at baseline than the survival group. To avoid Simpson’s paradox (which is a disproportionate allocation of some variables that can generate opposing and seemingly paradoxical results) [18], we adjusted all possible confounding factors that are reported to be associated with the severity of MAFLD and its outcomes. The Hazard ratio (HR) and 95% confidential interval (CI) of death outcomes in response to MAFLD across each PA subgroup were estimated. As the PA might have a interaction with HEI, we visualized the results of regression models with contour plots. We also used restricted cubic splines (RCS) to visualize the relationship between HEI scores and the risk of mortality in MAFLD. Statistical significance was set at *P* < 0.05. All analyses were performed using R software (https://www.r-project.org/).

## Results

### Baseline characteristics

In total, 19,599 participants were screened. After excluding cases without hepatic steatosis data (*n* = 6157), without other key data (*n* = 3030), and individuals without MAFLD (*n* = 6703), a total of 3709 participants with MAFLD were included in the final analysis (Supplementary Fig. 1). The mean age was 46.6 ± 15.4 years, and 49.7% of them were male. More than half of the patients had hypertension (59.9%) and 23.8% had diabetes mellitus. A total of 878 (23.7%) participants had low family incomes and 1691 (45.6%) had low educational levels (Table 1). The median follow-up duration was 26.2 (interquartile range 19.3–28.1) years. Among this population, 1549 (41.8%) deaths were recorded during the follow-up period. The comparison between survival and non-survival groups are shown in supplementary Table 1.

**Table 1:** The comparison of the baseline characteristics of the overall MAFLD patients.

Variables	Total
*N*	3709
Follow-up (years)	26.2 (19.3, 28.1)
Male, *n* (%)	1844 (49.7)
Age (years)	46.6 ± 15.4
Race, *n* (%)	
Non-Hispanic black	879 (23.7)
Other	2830 (76.3)
Low educational level	1691 (45.6)
Low family income	878 (23.7)
Overdrink, *n* (%)	213 (5.7)
Type 2 diabetes, *n* (%)	883 (23.8)
Hypertension, *n* (%)	2221 (59.9)
HEI score	63.0 (53.7, 72.2)
PA level	35.5 (3.5, 122.0)
Physical activity, *n* (%)	
Inactive	1847 (49.8)
Active	1862 (50.2)
BMI (kg/m^2^)	29.5 ± 6.3
WHR	1.0 ± 0.1
HbA1c (%)	5.8 ± 1.4
Cholesterol (mmol/L)	5.5 ± 1.2
Triglyceride (mmol/L)	2.1 ± 1.7
AST (U/L)	21 (17, 27)
ALT (U/L)	17 (12, 27)
eGFR (ml/min/1.73m^2^)	79.1 ± 18.7
FIB-4 scores	0.8 (0.6, 1.2)
NFS scores	−1.8 (−2.9, −0.6)

*MAFLD* metabolic (dysfunction-) associated fatty liver disease, *HEI* healthy Eating Index, *BMI* body mass index, *WHR* Waist hip ratio, *HbA1c* glycosylated hemoglobin; *ALT* alanine aminotransferase, *AST* aspartate aminotransferase, *eGFR* estimated glomerular filtration rate, *FIB-4* fibrosis 4 index; *NFS* NAFLD fibrosis score.

### Risk of all-cause mortality in overall patients

**Fig. 1:**
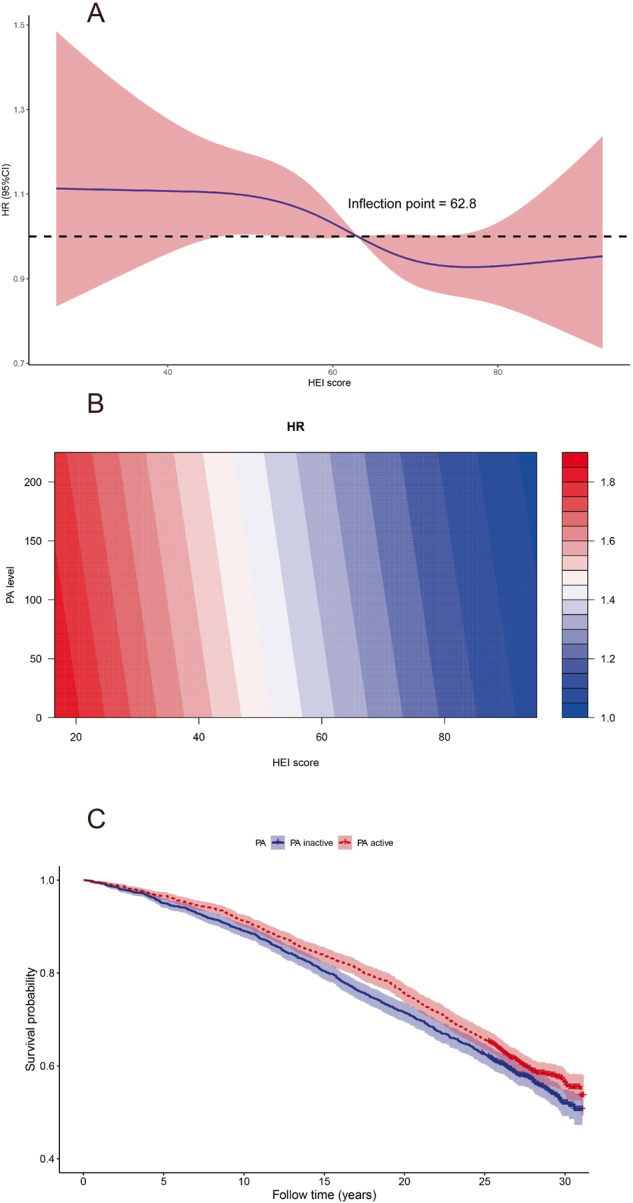
The association between HEI scores and the risk of all-cause mortality of MAFLD. **A** In overall population, restricted cubic splines showed the risk of all-cause mortality reduced with the increase of HEI scores. **B** Contour plot showed the risk of all-cause mortality in MAFLD patients with different HEI and PA. The color refers to the HR level. **C** Kaplan–Meier survival curves for all-cause mortality.

We used multivariate Cox regression to adjust for potential confounders of mortality, including race, age, sex, socioeconomic factors, demographic characteristics, diabetes history, hypertension history, metabolic profiles, renal function, liver enzymes, and liver fibrosis score. The results were presented in Table 2, showing both HEI score and PA level were inversely correlated with all-cause mortality (*P* < 0.05). The association between HEI scores and the risk of all-cause mortality of MAFLD were evaluated on a continuous scale with RCS based on multivariate Cox regressions (Fig. 1A), indicating the risk of mortality was inversely associated with HEI score and the inflection point of HEI score was 62.8. The contour plot (Fig. 1B) illustrated the interaction among diet quality, PA level, and mortality. The risk of all-cause mortality of the overall MAFLD patients decreased with the increase of HEI score and PA score.

**Table 2:** Cox regression of all-cause death in the overall MAFLD patients.

Variables	Univariate		Multivariate	
	HR (95% CI)	*P* value	HR (95% CI)	*P* value
HEI score	1.002 (0.999–1.006)	0.219	0.995 (0.992–0.999)	0.017
PA level	0.999 (0.999–1.000)	0.018	0.999 (0.999–1.000)	0.029
Male, *n* (%)	1.305 (1.181–1.442)	<0.001	1.156 (1.020–1.310)	0.024
Age (years)	1.081 (1.076–1.085)	<0.001	1.067 (1.06–1.074)	<0.001
Race, *n* (%)	1.042 (0.928–1.171)	0.485	1.228 (1.081–1.396)	0.002
Low educational level	1.662 (1.503–1.837)	<0.001	1.098 (0.983–1.226)	0.099
Low family income	0.916 (0.812–1.033)	0.153	1.128 (0.992–1.284)	0.066
Overdrink, *n* (%)	1.171 (0.956–1.434)	0.126	1.323 (1.072–1.633)	0.009
Type 2 diabetes, *n* (%)	2.691 (2.427–2.984)	<0.001	1.150 (1.005–1.315)	0.042
Hypertension, *n* (%)	2.405 (2.145–2.697)	<0.001	1.207 (1.070–1.361)	0.002
BMI (kg/m^2^)	1.009 (1.001–1.016)	0.027	0.996 (0.985–1.008)	0.509
WHR	46.218 (32.819–65.087)	<0.001	4.790 (2.303–9.963)	<0.001
HbA1c (%)	1.230 (1.200–1.260)	<0.001	1.103 (1.064–1.143)	<0.001
Cholesterol (mmol/L)	1.223 (1.182–1.266)	<0.001	0.997 (0.951–1.045)	0.892
Triglyceride (mmol/L)	1.075 (1.054–1.097)	<0.001	1.038 (1.006–1.071)	0.021
AST (U/L)	1.003 (1.001–1.006)	0.014	1.010 (1.005–1.015)	<0.001
ALT (U/L)	0.992 (0.989–0.995)	<0.001	0.991 (0.987–0.996)	<0.001
eGFR (ml/min/1.73m^2^)	0.960 (0.957–0.963)	<0.001	0.994 (0.990–0.998)	0.003
FIB-4 scores	1.443 (1.407–1.481)	<0.001	1.069 (0.961–1.189)	0.217
NFS scores	1.541 (1.497–1.587)	<0.001	1.021 (0.961–1.086)	0.500

*MAFLD* Metabolic (dysfunction-) associated fatty liver disease, *HEI* healthy Eating Index, *BMI,* body mass index, *WHR* Waist hip ratio, *HbA1c* glycosylated hemoglobin, *ALT* alanine aminotransferase, *AST* aspartate aminotransferase, *eGFR* estimated glomerular filtration rate, *FIB-4* fibrosis 4 index; *NFS* NAFLD fibrosis score.

### Group by PA

The total MAFLD patients were divided into two subgroups according to the median value of PA scores (PA active group vs. PA inactive group). A comparison of baseline characteristics between the groups of patients with PA active and inactive is shown in Table 3. Compared to PA inactive group, PA active group had more male patients (56.4% vs. 43.0%, *P* < 0.001), lower educational level (35.2% vs. 56.0%, *P* < 0.001) and low family income (18.6% vs. 28.8%, *P* < 0.001). As expected, BMI were lower in PA active group (28.9 ± 5.9 kg/m^2^ vs. 30.2 ± 6.7 kg/m^2^, *P* < 0.001). The metabolic derangement (diabetes, WHR, and HbA1c) were less severe in active individuals than in inactive individuals (all *P* < 0.05). Patients in PA active group tended to eat better than those in the PA inactive group (HEI score 64.4[54.2, 73.5 vs. 61.9 [53.3, 71.1], *P* < 0.001). The Kaplan–Meier curve showed active PA levels was associated with better prognosis of MAFLD (log-rank *P* = 0.024, Fig. 1C).

**Table 3:** The comparison between PA inactive and active groups.

Variables	PA		*P* value
	inactive	active	
*N*	1847	1862	–
HEI score	61.9 (53.3, 71.1)	64.4 (54.2, 73.5)	<0.001
PA level	3.5 (0, 17.0)	120.3 (70.0, 193.3)	<0.001
Death, *n* (%)	799 (43.3)	750 (40.3)	0.071
Follow-up (years)	26.0 (17.8, 28.1)	26.3 (20.4, 28.2)	0.007
Male, *n* (%)	794 (43.0)	1050 (56.4)	<0.001
Age (years)	46.7 ± 15.1	46.6 ± 15.6	0.869
Race, *n* (%)			0.43
Non-Hispanic black	427 (23.1)	452 (24.3)	–
Other	1420 (76.9)	1410 (75.7)	–
Low educational level	1035 (56.0)	656 (35.2)	<0.001
Low family income	532 (28.8)	346 (18.6)	<0.001
Overdrink, *n* (%)	105 (5.7)	108 (5.8)	0.936
Type 2 diabetes, *n* (%)	495 (26.8)	388 (20.8)	<0.001
Hypertension, *n* (%)	1112 (60.2)	1109 (59.6)	0.713
BMI (kg/m^2^)	30.2 ± 6.7	28.9 ± 5.9	<0.001
WHR	1.0 ± 0.1	0.9 ± 0.1	0.002
HbA1c (%)	5.9 ± 1.5	5.7 ± 1.3	<0.001
Cholesterol (mmol/L)	5.4 ± 1.2	5.5 ± 1.2	0.186
Triglyceride (mmol/L)	2.1 ± 1.6	2.0 ± 1.8	0.497
AST (U/L)	20 (16, 26)	21 (17, 28)	<0.001
ALT (U/L)	17 (12, 26)	18 (12, 27)	0.062
eGFR (ml/min/1.73m^2^)	79.9 ± 19.1	78.3 ± 18.3	0.008
FIB-4 scores	0.8 (0.5, 1.2)	0.9 (0.6, 1.3)	0.001
NFS scores	−1.8 (−2.9, −0.6)	−1.9 (−2.9, −0.7)	0.299

*MAFLD* Metabolic (dysfunction-) associated fatty liver disease, *HEI* healthy Eating Index, *BMI* body mass index, *WHR* Waist hip ratio, *HbA1c* glycosylated hemoglobin, *ALT* alanine aminotransferase, *AST* aspartate aminotransferase, *eGFR* estimated glomerular filtration rate, *FIB-4* fibrosis 4 index, *NFS* NAFLD fibrosis score.

### Subgroup analysis according to physical activity

**Fig. 2:**
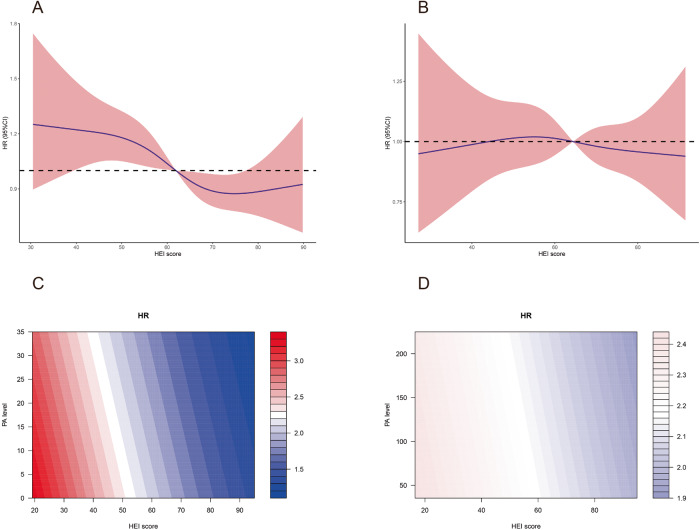
The association between HEI scores and the risk of all-cause mortality of MAFLD grouped by PA. **A** In PA inactive group, restricted cubic splines showed the HR for all-cause mortality reduced with the increase of HEI scores. **B** In PA active group, restricted cubic splines showed the HR for all-cause mortality was not significantly changed over HEI scores. **C** Contour plot of PA inactive group. **D** Contour plot of PA active group. The color refers to the HR level. **C** and **D** showed the difference of HRs was significant in PA inactive group but not the active group.

Table 4 shows the results of multivariate Cox regression in different PA subgroups after adjustment for confounding factors. In PA inactive patients, HEI score was inversely correlated with all-cause mortality (HR = 0.992, 95% CI: 0.986–0.997, *P* = 0.002), whereas no statistically significant association between HEI score and all-cause mortality was found in the PA active subgroup (HR = 0.999, 95% CI: 0.994–1.004, *P* = 0.672). These results were shown in Table 4 and visualized by RCS (Fig. 2A, B). Contour plots also illustrated an inversely correlation between HEI score and all-cause mortality in PA inactive MAFLD, but not in PA active population (Fig. 2C, D).

**Table 4:** Cox regression of all-cause death in the overall MAFLD patients grouped by PA level.

Variables	PA inactive		PA active	
	HR (95% CI)	*P* value	HR (95% CI)	*P* value
HEI score	0.992 (0.986–0.997)	0.002	0.999 (0.994–1.004)	0.672
PA level	0.993 (0.986–1.000)	0.043	1.000 (0.999–1.001)	0.948
Male, *n* (%)	1.220 (1.022–1.455)	0.027	1.125 (0.937–1.352)	0.207
Age (years)	1.063 (1.053–1.073)	<0.001	1.070 (1.060–1.080)	<0.001
Race, *n* (%)	1.160 (0.974–1.382)	0.097	1.261 (1.041–1.527)	0.018
Low educational level	1.170 (0.999–1.370)	0.051	1.012 (0.863–1.188)	0.880
Low family income	1.017 (0.861–1.201)	0.848	1.264 (1.026–1.558)	0.028
Overdrink, *n* (%)	1.426 (1.065–1.910)	0.017	1.253 (0.918–1.709)	0.155
Type 2 diabetes, *n* (%)	1.222 (1.013–1.475)	0.036	1.069 (0.881–1.297)	0.501
Hypertension, *n* (%)	1.245 (1.049–1.479)	0.012	1.200 (1.012–1.423)	0.036
BMI (kg/m^2^)	1.003 (0.988–1.019)	0.685	0.986 (0.968–1.004)	0.123
WHR	5.234 (1.989–13.770)	0.001	3.582 (1.132–11.328)	0.030
HbA1c (%)	1.078 (1.027–1.132)	0.003	1.145 (1.087–1.207)	0.000
Cholesterol (mmol/L)	1.009 (0.943–1.081)	0.788	0.995 (0.931–1.064)	0.890
Triglyceride (mmol/L)	1.065 (1.016–1.117)	0.009	1.018 (0.970–1.067)	0.472
AST (U/L)	1.019 (1.009–1.029)	<0.001	1.007 (1.000–1.013)	0.038
ALT (U/L)	0.984 (0.976–0.992)	<0.001	0.995 (0.988–1.001)	0.107
eGFR (ml/min/1.73m^2^)	0.992 (0.987–0.997)	0.003	0.996 (0.990–1.002)	0.177
FIB-4 scores	0.985 (0.801–1.212)	0.888	1.130 (0.991–1.288)	0.067
NFS scores	1.021 (0.933–1.116)	0.657	1.026 (0.938–1.122)	0.582

*MAFLD* Metabolic (dysfunction-) associated fatty liver disease, *HEI* healthy Eating Index, *BMI* body mass index, *WHR* Waist hip ratio, *HbA1c* glycosylated hemoglobin, *ALT* alanine aminotransferase, *AST* aspartate aminotransferase, *eGFR* estimated glomerular filtration rate; *FIB-4* fibrosis 4 index, *NFS* NAFLD fibrosis score.

### Cause-specific mortality

In overall population, HEI score and PA level were not correlated with cardiovascular-related death and cancer-related death after adjustment for potential confounders (supplementary Table 2). However, in subgroups analyses, HEI score was inversely related with both cardiovascular-related death (HR = 0.988, 95% CI: 0.978–0.999, *P* = 0.027) and cancer-related death (HR = 0.986, 95% CI: 0.975–0.998, *P* = 0.018) in PA inactive MAFLD, while no statistically significant association between HEI score and cause-specific mortality was detected in PA active group (supplementary Tables 3 and 4).

## Discussion

Lifestyle modification, including dietary management and increased physical activity, are currently effective interventions for MAFLD. In this cohort study, we found that a healthy diet improved the survival of MAFLD individuals with low PA levels. However, dietary quality did not add additional benefit regarding long-term survival to those with active PA levels. This result indicates that healthy diet and physical activity may have various impact as lifestyle interventions for MAFLD. The importance of a high-quality diet should be emphasized, especially for individuals with MAFLD and sedentary lifestyles, regarding long-term prognosis.

We found that in patients with moderate or active PA levels, lower dietary quality did not significantly affect mortality. This finding has been supported by previous studies, in which researchers found that exercise can mitigate the harmful effects of poor-quality food consumption. Aerobic exercise training has been reported to prevent endothelial dysfunction in healthy young men consuming sugar-sweetened beverages [19]. Similarly, high-intensity exercise has been shown to offset the deleterious effects of 14 days of fast-food meals [20], excess energy intake [21], or a high-fructose diet [22]. The harm of low diet quality may be offset by higher exercise levels; therefore, for individuals who have already been physically active, diet quality seems to be less essential.

However, in physically inactive individuals, diet is a major determinant of long-term outcomes. It is noteworthy that at baseline, patients with higher HEI scores were older and had more metabolic dysfunction, which were all unfavorable factors for survival. Interestingly, however, they also had a lower mortality rate than those with lower dietary quality. It has been reported that a low-quality diet is associated with oxidative stress and high levels of pro-inflammatory biomarkers [23]. The dietary quality score is inversely associated with the risk of frailty and mortality in adults [24]. A meta-analysis of 12 cohort studies and one cross-sectional study showed that strict adherence to a high-quality diet reduced the risks of all-cause and cause-specific mortality [25]. Therefore, individualized dietary management is important for patients with MAFLD.

A meta-analysis of 20 randomized control trials and 1073 patients with NAFLD showed that exercise alone or combined with dietary intervention improved serum levels of liver enzymes and liver fat or histology [26]. According to the results of this study, active exercise is important for individuals with MAFLD, but for those who cannot maintain a physical exercise routine, a high-quality diet is strongly recommended to reduce the future mortality risk. Therefore, adherence to at least one approach, being active or having a good quality diet, may offer the benefits of lifestyle modifications.

We found that high dietary quality may reduce the risk of mortality in patients with MAFLD. However, Yoo et al. failed to find an association between dietary quality and lower mortality risk in NAFLD [27]. This discrepancy may be due to the different definitions of fatty liver disease between the studies (MAFLD vs. NAFLD) and the adjustments for PA levels in the present study. As mentioned above, exercise may supersede the deleterious mortality effects of low-quality diets.

In this study, although all participants were diagnosed with MAFLD, some had active exercise levels and high-quality diets, which was counterintuitive at first glance. It should be noted that PA and diet information were obtained at the time of the survey, and those patients may have already been diagnosed with fatty liver disease or other metabolic syndromes and thus, had increased awareness of lifestyle modifications. We only analyzed exercise and dietary habits at the beginning of follow-up. Lifestyle patterns could have changed over time. A well-designed longitudinal cohort study is needed to verify our findings.

The strengths of this study are the nationally representative cohort and follow-up duration of >25 years to assess mortality in a large number of individuals. Thus, our findings can be generalized. However, our study had several limitations. First, diet and PA were self-reported, which can mean reporting and recall biases. Second, the HEI score was calculated using the 24-hour dietary recall. The limitations of this method for measuring dietary quality are well recognized. However, it is adequate for grouping participants into various levels of diet quality. Third, the American College of Sports Medicine recommends moderate- and vigorous-intensity exercise to achieve a total energy expenditure of ≥500-1000 MET·min·wk [11]. In this study, we were not able to calculate the exact energy expenditure of each participant as the time duration of each exercise session was not precisely defined in the original database. To solve this problem, we simply divided study population into two group according to the median calculated-PA scores. This might inevitably lead to missed classification of some patients. Finally, the assessments of PA and diet information rely solely on self-administered questionnaires, which could easily result in recall bias. More objective evaluation methods, such as detailed food diaries and wearable activity trackers, are required in future study.

In conclusion, a high-quality diet may improve the survival rate of patients with low PA levels. The quality of diet assessed by the HEI score did not significantly influence the mortality of MAFLD patients with moderate to active PA levels. The importance of a high-quality diet should be emphasized, especially for individuals with MAFLD and sedentary lifestyles.

### Supplementary information

supplementary material legends

Supplementary Table 1

Supplementary Table 2

Supplementary Table 3

Supplementary Table 4

Supplementary Figure 1

## Data Availability

Publicly available datasets were analyzed in this study. The raw data are available from National Health and Nutrition Examination Survey program (https://www.cdc.gov/nchs/nhanes/index.htm).
