# Supplementary material for: The prognostic role of diet quality in patients with MAFLD and physical activity: data from NHANES
Source: Nutr Diabetes. 2024 Feb 23;14:4. doi: 10.1038/s41387-024-00261-x (PMC10891170; doi:10.1038/s41387-024-00261-x)
Supplement: Supplementary file 1 — supplementary material legends [file 41387_2024_261_MOESM1_ESM.doc]

**Supplementary figures**

Supplementary Figure 1 Flowchart of case selection

**Supplementary Tables**

Supplementary Table 1 The comparison of the baseline characteristics between the survival group and death group

Supplementary Table 2 Cox multivariate regression of cardiovascular-related mortality and cancer-related mortality in the overall patients

Supplementary Table 3 Cox multivariate regression of cardiovascular-related mortality grouped by PA

Supplementary Table 4 Cox multivariate regression to adjust for potential confounders of cancer-related mortality grouped by PA
