# Supplementary material for: The prognostic role of diet quality in patients with MAFLD and physical activity: data from NHANES
Source: Nutr Diabetes. 2024 Feb 23;14:4. doi: 10.1038/s41387-024-00261-x (PMC10891170; doi:10.1038/s41387-024-00261-x)
Supplement: Supplementary file 2 — Supplementary Table 1 [file 41387_2024_261_MOESM2_ESM.doc]

Supplementary Table 1 The comparison of the baseline characteristics between the survival group and death group

| Variables | Survival | All-cause Death | *P* |
| --- | --- | --- | --- |
| N | 2160 | 1549 |  |
| Follow-up (years) | 27.6 (26.4, 29.3) | 16.8 (10.5, 22.6) | < 0.001 |
| Male, n (%) | 996 (46.1) | 848 (54.7) | < 0.001 |
| Age (years) | 38.8 ± 11.9 | 57.5 ± 12.9 | < 0.001 |
| Race, n (%) |  |  | 0.511 |
| Non-Hispanic black | 503 (23.3) | 376 (24.3) |  |
| Other | 1657 (76.7) | 1173 (75.7) |  |
| Low educational level | 841 (38.9) | 850 (54.9) | < 0.001 |
| Low family income | 536 (24.8) | 342 (22.1) | 0.058 |
| Overdrink, n (%) | 113 (5.2) | 100 (6.5) | 0.131 |
| Type 2 diabetes, n (%) | 297 (13.8) | 586 (37.8) | < 0.001 |
| Hypertension, n (%) | 1065 (49.3) | 1156 (74.6) | < 0.001 |
| HEI score | 62.9 (53.9, 71.5) | 63.2 (53.3, 73.6) | 0.273 |
| PA level | 40.0 (5.0, 124.1) | 31.5 (0, 117.5) | 0.013 |
| Physical activity, n (%) |  |  | 0.071 |
| Inactive | 1048 (48.5) | 799 (51.6) |  |
| Active | 1112 (51.5) | 750 (48.4) |  |
| BMI (kg/m^2^) | 29.3 ± 6.3 | 29.8 ± 6.3 | 0.010 |
| WHR | 0.9 ± 0.1 | 1.0 ± 0.1 | < 0.001 |
| HbA1c (%) | 5.5 ± 1.1 | 6.2 ± 1.7 | < 0.001 |
| Cholesterol (mmol/L) | 5.3 ± 1.1 | 5.7 ± 1.3 | < 0.001 |
| Triglyceride (mmol/L) | 1.9 ± 1.5 | 2.2 ± 1.9 | < 0.001 |
| AST (U/L) | 21 (17, 27) | 20 (17, 27) | 0.950 |
| ALT (U/L) | 19 (13, 28) | 16 (12, 23) | < 0.001 |
| eGFR (ml/min/1.73m^2^) | 85.1 ± 16.3 | 70.8 ± 18.6 | < 0.001 |
| FIB-4 scores | 0.7 (0.5, 0.9) | 1.2 (0.8, 1.6) | < 0.001 |
| NFS scores | -2.5 (-3.3, -1.5) | -0.8 (-1.9, 0.1) | < 0.001 |

Abbreviations: HEI, healthy Eating Index; BMI, body mass index; WHR, Waist hip ratio; HbA1c, glycosylated hemoglobin; ALT, alanine aminotransferase; AST, aspartate aminotransferase; eGFR, estimated glomerular filtration rate; FIB-4, fibrosis 4 index; NFS, NAFLD fibrosis score.
