# Supplementary material for: The prognostic role of diet quality in patients with MAFLD and physical activity: data from NHANES
Source: Nutr Diabetes. 2024 Feb 23;14:4. doi: 10.1038/s41387-024-00261-x (PMC10891170; doi:10.1038/s41387-024-00261-x)
Supplement: Supplementary file 3 — Supplementary Table 2 [file 41387_2024_261_MOESM3_ESM.doc]

Supplementary Table 2 Cox multivariate regression of cardiovascular-related mortality and cancer-related mortality in the overall patients

| Variables | Cardiovascular-related mortality | | Cancer-related mortality | |
| --- | --- | --- | --- | --- |
|  | HR (95% CI) | *P* | HR (95% CI) | *P* |
| HEI score | 0.993 (0.986–1.000) | 0.060 | 0.993 (0.985–1.001) | 0.088 |
| PA level | 1.000 (0.999–1.001) | 0.777 | 1.000 (0.998–1.001) | 0.480 |
| Male, n (%) | 1.572 (1.218–2.028) | 0.001 | 1.066 (0.823–1.379) | 0.629 |
| Age (years) | 1.073 (1.059–1.087) | <0.001 | 1.054 (1.041–1.068) | <0.001 |
| Race, n (%) | 1.123 (0.873–1.444) | 0.368 | 1.161 (0.886–1.521) | 0.278 |
| Low educational level | 1.116 (0.900–1.384) | 0.318 | 1.123 (0.890–1.415) | 0.328 |
| Low family income | 1.230 (0.959–1.578) | 0.103 | 1.082 (0.825–1.420) | 0.569 |
| Overdrink, n (%) | 1.180 (0.759–1.835) | 0.463 | 1.586 (1.060–2.371) | 0.025 |
| Type 2 diabetes, n (%) | 1.082 (0.828–1.412) | 0.564 | 0.873 (0.648–1.176) | 0.372 |
| Hypertension, n (%) | 1.455 (1.135–1.866) | 0.003 | 1.051 (0.826–1.337) | 0.687 |
| BMI (kg/m^2^) | 1.014 (0.990–1.037) | 0.254 | 0.967 (0.943–0.991) | 0.007 |
| WHR | 2.070 (0.469–9.139) | 0.337 | 6.256 (1.396–28.04) | 0.017 |
| HbA1c (%) | 1.154 (1.082–1.230) | <0.001 | 0.997 (0.903–1.100) | 0.949 |
| Cholesterol (mmol/L) | 1.039 (0.949–1.138) | 0.412 | 0.922 (0.832–1.022) | 0.122 |
| Triglyceride (mmol/L) | 1.064 (1.007–1.125) | 0.026 | 1.030 (0.957–1.109) | 0.434 |
| AST (U/L) | 1.011 (0.994–1.028) | 0.197 | 1.008 (0.997–1.018) | 0.156 |
| ALT (U/L) | 0.984 (0.972–0.997) | 0.016 | 0.992 (0.982–1.002) | 0.125 |
| eGFR (ml/min/1.73m^2^) | 0.986 (0.979–0.994) | <0.001 | 0.998 (0.990–1.006) | 0.607 |
| FIB-4 scores | 0.813 (0.565–1.169) | 0.263 | 1.015 (0.811–1.270) | 0.895 |
| NFS scores | 1.040 (0.902–1.199) | 0.593 | 1.112 (0.979–1.262) | 0.102 |

Abbreviations: HEI, healthy Eating Index; BMI, body mass index; WHR, Waist hip ratio; HbA1c, glycosylated hemoglobin; ALT, alanine aminotransferase; AST, aspartate aminotransferase; eGFR, estimated glomerular filtration rate; FIB-4, fibrosis 4 index; NFS, NAFLD fibrosis score.
