# Supplementary material for: The prognostic role of diet quality in patients with MAFLD and physical activity: data from NHANES
Source: Nutr Diabetes. 2024 Feb 23;14:4. doi: 10.1038/s41387-024-00261-x (PMC10891170; doi:10.1038/s41387-024-00261-x)
Supplement: Supplementary file 4 — Supplementary Table 3 [file 41387_2024_261_MOESM4_ESM.doc]

Supplementary Table 3 Cox multivariate regression of cardiovascular-related mortality grouped by PA

| Variables | PA inactive | | PA active | |
| --- | --- | --- | --- | --- |
|  | HR (95% CI) | *P* | HR (95% CI) | *P* |
| HEI score | 0.988 (0.978–0.999) | 0.027 | 0.997 (0.987–1.007) | 0.595 |
| PA level | 0.996 (0.982–1.010) | 0.531 | 1.000 (0.998–1.002) | 0.884 |
| Male, n (%) | 1.539 (1.074–2.203) | 0.019 | 1.666 (1.147–2.419) | 0.007 |
| Age (years) | 1.068 (1.048–1.088) | 0.000 | 1.075 (1.055–1.096) | 0.000 |
| Race, n (%) | 0.958 (0.67–1.370) | 0.816 | 1.358 (0.940–1.960) | 0.103 |
| Low educational level | 1.245 (0.904–1.715) | 0.179 | 1.036 (0.766–1.400) | 0.821 |
| Low family income | 1.108 (0.798–1.539) | 0.541 | 1.317 (0.889–1.953) | 0.170 |
| Overdrink, n (%) | 1.784 (1.028–3.098) | 0.040 | 0.754 (0.349–1.631) | 0.473 |
| Type 2 diabetes, n (%) | 1.129 (0.779–1.638) | 0.522 | 1.098 (0.749–1.612) | 0.631 |
| Hypertension, n (%) | 1.612 (1.114–2.333) | 0.011 | 1.385 (0.988–1.943) | 0.059 |
| BMI (kg/m^2^) | 1.017 (0.986–1.049) | 0.297 | 1.009 (0.973–1.047) | 0.630 |
| WHR | 2.082 (0.304–14.288) | 0.455 | 1.958 (0.189–20.329) | 0.574 |
| HbA1c (%) | 1.079 (0.979–1.189) | 0.127 | 1.229 (1.131–1.335) | 0.000 |
| Cholesterol (mmol/L) | 0.960 (0.836–1.102) | 0.558 | 1.107 (0.983–1.246) | 0.094 |
| Triglyceride (mmol/L) | 1.087 (0.990–1.195) | 0.081 | 1.059 (0.979–1.147) | 0.155 |
| AST (U/L) | 1.025 (1.002–1.049) | 0.033 | 0.983 (0.947–1.019) | 0.345 |
| ALT (U/L) | 0.979 (0.960–0.998) | 0.027 | 0.993 (0.971–1.015) | 0.517 |
| eGFR (ml/min/1.73m^2^) | 0.984 (0.974–0.995) | 0.003 | 0.988 (0.977–0.999) | 0.027 |
| FIB-4 scores | 0.683 (0.394–1.183) | 0.174 | 1.216 (0.697–2.120) | 0.491 |
| NFS scores | 1.117 (0.916–1.362) | 0.273 | 0.902 (0.729–1.117) | 0.346 |

Abbreviations: HEI, healthy Eating Index; BMI, body mass index; WHR, Waist hip ratio; HbA1c, glycosylated hemoglobin; ALT, alanine aminotransferase; AST, aspartate aminotransferase; eGFR, estimated glomerular filtration rate; FIB-4, fibrosis 4 index; NFS, NAFLD fibrosis score.
