# Supplementary material for: The prognostic role of diet quality in patients with MAFLD and physical activity: data from NHANES
Source: Nutr Diabetes. 2024 Feb 23;14:4. doi: 10.1038/s41387-024-00261-x (PMC10891170; doi:10.1038/s41387-024-00261-x)
Supplement: Supplementary file 5 — Supplementary Table 4 [file 41387_2024_261_MOESM5_ESM.doc]

Supplementary Table 4 Cox multivariate regression to adjust for potential confounders of cancer-related mortality grouped by PA

| Variables | PA inactive | | PA active | |
| --- | --- | --- | --- | --- |
|  | HR (95% CI) | *P* | HR (95% CI) | *P* |
| HEI score | 0.986 (0.975–0.998) | 0.018 | 0.998 (0.986–1.009) | 0.660 |
| PA level | 0.983 (0.968–0.999) | 0.034 | 1.001 (0.998–1.003) | 0.637 |
| Male, n (%) | 1.123 (0.775–1.628) | 0.540 | 1.035 (0.716–1.496) | 0.854 |
| Age (years) | 1.040 (1.021–1.060) | <0.001 | 1.065 (1.045–1.086) | <0.001 |
| Race, n (%) | 1.091 (0.749–1.588) | 0.651 | 1.263 (0.850–1.878) | 0.248 |
| Low educational level | 1.142 (0.821–1.589) | 0.430 | 1.115 (0.801–1.553) | 0.519 |
| Low family income | 0.936 (0.658–1.332) | 0.713 | 1.200 (0.778–1.851) | 0.410 |
| Overdrink, n (%) | 1.868 (1.105–3.158) | 0.020 | 1.356 (0.717–2.564) | 0.350 |
| Type 2 diabetes, n (%) | 1.113 (0.741–1.673) | 0.607 | 0.698 (0.449–1.086) | 0.111 |
| Hypertension, n (%) | 1.171 (0.825–1.662) | 0.378 | 0.952 (0.679–1.333) | 0.773 |
| BMI (kg/m^2^) | 0.978 (0.946–1.010) | 0.171 | 0.956 (0.919–0.994) | 0.025 |
| WHR | 6.66 (0.828–53.594) | 0.075 | 6.220 (0.696–55.59) | 0.102 |
| HbA1c (%) | 0.955 (0.836–1.090) | 0.491 | 1.061 (0.917–1.228) | 0.426 |
| Cholesterol (mmol/L) | 1.009 (0.874–1.165) | 0.904 | 0.853 (0.734–0.992) | 0.039 |
| Triglyceride (mmol/L) | 1.080 (0.982–1.187) | 0.115 | 0.963 (0.844–1.098) | 0.571 |
| AST (U/L) | 1.014 (0.997–1.031) | 0.117 | 1.005 (0.986–1.025) | 0.593 |
| ALT (U/L) | 0.987 (0.972–1.003) | 0.104 | 0.991 (0.974–1.008) | 0.296 |
| eGFR (ml/min/1.73m^2^) | 0.997 (0.986–1.008) | 0.596 | 0.997 (0.985–1.010) | 0.649 |
| FIB-4 scores | 1.176 (0.838–1.652) | 0.349 | 0.961 (0.659–1.401) | 0.835 |
| NFS scores | 1.073 (0.901–1.277) | 0.432 | 1.110 (0.914–1.348) | 0.295 |

Abbreviations: HEI, healthy Eating Index; BMI, body mass index; WHR, Waist hip ratio; HbA1c, glycosylated hemoglobin; ALT, alanine aminotransferase; AST, aspartate aminotransferase; eGFR, estimated glomerular filtration rate; FIB-4, fibrosis 4 index; NFS, NAFLD fibrosis score.
