# Supplementary figures and images for: The prognostic role of diet quality in patients with MAFLD and physical activity: data from NHANES
Source: Nutr Diabetes. 2024 Feb 23;14:4. doi: 10.1038/s41387-024-00261-x (PMC10891170; doi:10.1038/s41387-024-00261-x)

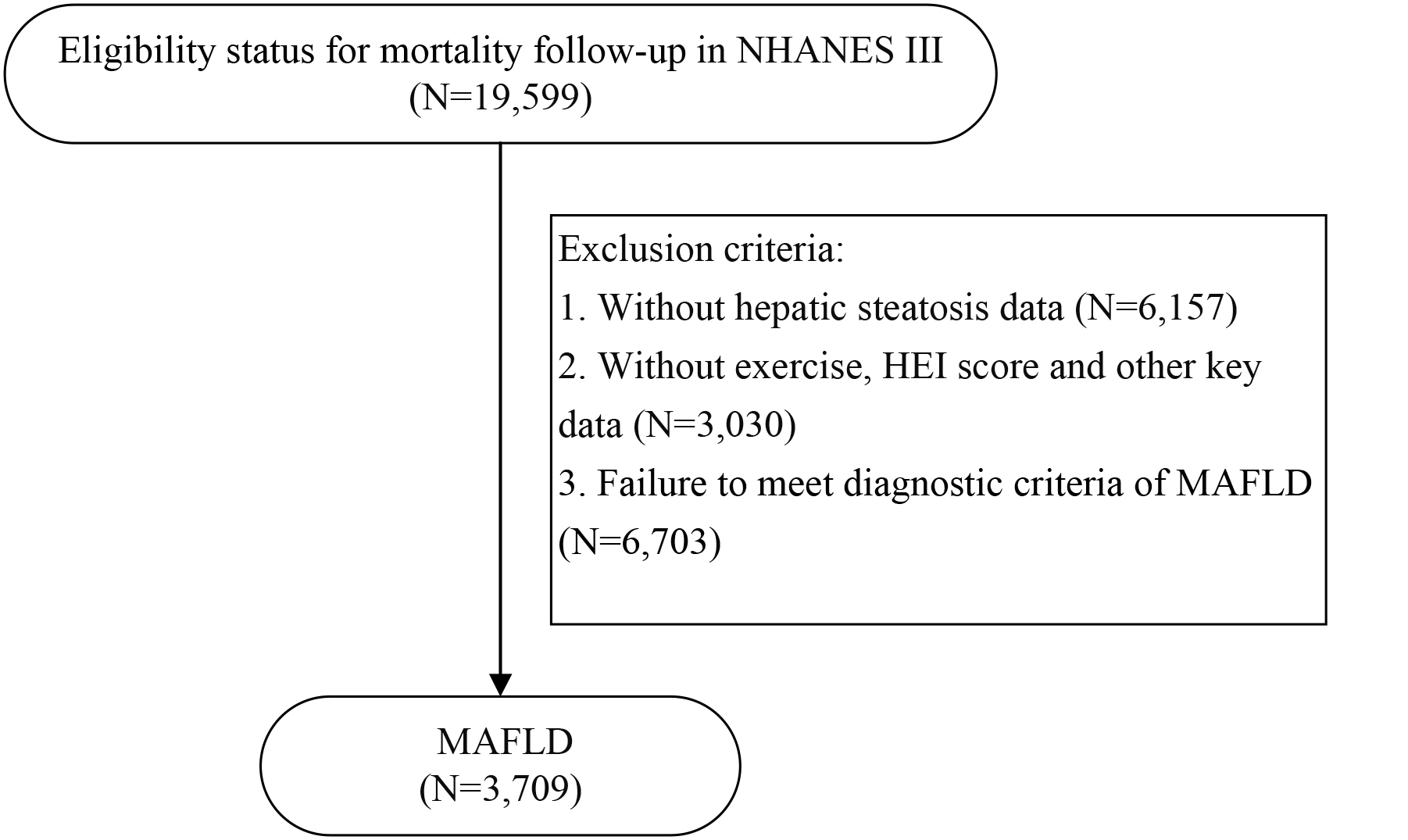

Supplement: Supplementary file 6 — Supplementary Figure 1 [file 41387_2024_261_MOESM6_ESM.jpg]
